# Hepatoprotective Effect of *Lactobacillus plantarum* HFY09 on Ethanol-Induced Liver Injury in Mice

**DOI:** 10.3389/fnut.2021.684588

**Published:** 2021-06-24

**Authors:** Yi Gan, Jin Tong, Xianrong Zhou, Xingyao Long, Yanni Pan, Weiwei Liu, Xin Zhao

**Affiliations:** ^1^Chongqing Collaborative Innovation Center for Functional Food, Chongqing University of Education, Chongqing, China; ^2^Chongqing Engineering Research Center of Functional Food, Chongqing University of Education, Chongqing, China; ^3^Chongqing Engineering Laboratory for Research and Development of Functional Food, Chongqing University of Education, Chongqing, China; ^4^Department of Gastroenterology and Hepatology, Chongqing Emergency Medical Center, Chongqing University Central Hospital, Chongqing, China; ^5^School of Public Health and Management, Chongqing Medical University, Chongqing, China

**Keywords:** lactic acid bacteria, alcoholic liver injury, hepatoprotective effect, antioxidant, anti-inflammatory

## Abstract

*Lactobacillus plantarum* is a bacterial strain that is used as a probiotic with health-promoting effects. Our study investigated the hepatoprotective effect of *Lactobacillus plantarum* HFY09 (LP-HFY09) in mice with ethanol-induced liver injury. The protection afforded by LP-HFY09 was evaluated by observing the morphology of hepatic tissue and measuring liver lipid indexes and function indexes, levels of anti-oxidative enzymes, and anti-inebriation enzymes, as well as oxidative metabolism-related gene expression. Gavage administration of LP-HFY09 [1 × 10^9^ CFU/kg body weight (bw)] limited the loss of bw, alcohol damage to the liver, and maintained the normal hepatic tissue morphology. *Lactobacillus plantarum* HFY09 intervention in ethanol-induced mice led to decreases in serum triglyceride (TG), total cholesterol (TC), aspartic transaminase, alanine transaminase, hyaluronidase (HAase), and precollagen III (PC III), and increases in liver alcohol dehydrogenase (ADH), and acetaldehyde dehydrogenase (ALDH). *Lactobacillus plantarum* HFY09 assisted with alleviating inflammation by elevating the level of interleukin 10 (IL-10) and decreasing the levels of pro-inflammatory factors [IL-6, IL-1β, and tumor necrosis factor-α (TNF)-α]. *Lactobacillus plantarum* HFY09 significantly elevated hepatic levels of superoxide dismutase (SOD) and glutathione (GSH), and decreased liver malondialdehyde (MDA) from 3.45 to 1.64 nmol/mg protein. *Lactobacillus plantarum* HFY09 exhibited an overall strong regulatory effect on liver protection when compared to that of commercial *Lactobacillus delbrueckii* subsp. *bulgaricus*. The hepatoprotective effect of LP-HFY09 was reflected by the upregulated expression of peroxisome proliferator activated-receptors α, SOD1, SOD2, glutathione peroxidase (GSH-Px), nicotinamide adenine dinucleotide phosphate (NADPH), and catalase (CAT), and the downregulated expression of cyclooxygenase-1 (COX1), c-Jun N-terminal kinase (JNK), and extracellular regulated protein kinases (ERK). Administration of LP-HFY09 at a concentration of 1.0 × 10^9^ CFU/kg bw could be a potential intervention, for people who frequently consume alcohol.

## Introduction

Alcoholic beverages, including distilled spirits, fermented wine, and mixed wine, have been consumed for thousands of years. However, ethanol is oxidized by alcohol dehydrogenase (ADH) to acetaldehyde, which is the predominant intermediate toxic product of glycolysis. Alcohol consumption can also lead to liver injury and hepatocyte death via apoptotic and necroptotic pathways (1). Alcoholic beverages may have side effects on overall health that are responsible for 3.6% of worldwide cancer cases and 20% of alcoholic liver disease (ALD) cases (1, 2). Alcoholic liver disease is a form of liver damage encompassing a spectrum of phenotypes including steatohepatitis, cirrhosis, progressive fibrosis, and hepatocellular carcinoma, and it is primarily caused by excessive alcohol consumption (3, 4). Oxidative stress and inflammatory milieu play important roles in the pathogenesis of this disease.

After ethanol intake, oxidative stress occurs in the liver due to reactive oxygen species (ROS)-damaged hepatocytes, gut endotoxin-activated Kupffer cells, and leukocyte infiltration (5, 6). Ethanol also increases the mitochondrial ROS by inducing dysfunction of electron transport chain proteins, which leads to further aggravation of mitochondrial dysfunction (7). Ethanol-induced oxidative stress also decreases the mitochondrial level of reduced glutathione (GSH) in the liver by inhibiting the transportation of GSH through the mitochondrial membrane (8). Moreover, TNF-α is a critical etiological factor with respect to liver damage caused by alcohol (9).

Lactic acid bacteria (LAB) with good acid tolerance ability are an important group of gram-positive microorganisms that have been used in the food industry. Some species of LAB have been confirmed to confer health-protective effects against various disorders (10). Studies also shown that oral administration of LAB could be used in the treatment of alcohol-based liver damage via its multi-target action without side effects (11, 12). These probiotic bacteria mainly increase the antioxidative and anti-inflammatory capacities of ethanol-induced liver-injury rats (13, 14). Moreover, *Lactobacillus* strains can prevent hepatic steatosis and injury from chronic alcohol exposure (15).

The current study appraised the hepatic protection capacity and anti-oxidant regulatory effect of *Lactobacillus plantarum* HFY09 (LP-HFY09). We measured its antioxidant characteristics, inflammatory level, and mRNA expression of antioxidant-related genes in ethanol-fed mice, aiming to provide the data that will support the development of newly isolated probiotics.

## Materials and Methods

### Experimental Strain

*Lactobacillus plantarum* HFY09 was isolated from natural fermented milk in Sichuan, China. It has been identified in the NCBI's database by the Basic Local Alignment Search Tool, and preserved (CGMCC No. 16633). A commercial LAB strain [Lactobacillus delbrueckii subsp. Bulgaricus (LDSB), CGMCC No. 1.16075] was used as a comparison. Before using, both bacterial strains were activated, collected, centrifuged (3,000 rpm, 10 min), and then resuspended in sterile saline.

### Animal Models of Ethanol-Induced Liver-Injury

Forty male Kunming mice (6 weeks old) (16) were purchased from the Experimental Animal Center of Chongqing Medical University (Chongqing, China). They were acclimated to room temperature (25 ± 2°C), moderate relative humidity (55 ± 5%), and a 12-h day/12-h night cycle, with food and water provided ad libitum for 7 days. Then, they were randomly divided into four groups (n = 10): (i) control group, (ii) EtOH fed group, (iii) EtOH fed + LP-HFY09, and (iv) EtOH fed + LDSB.

Mice in all groups were fed with a normal diet, and the body weight (bw) were recorded daily. On the first 7 days, the mice in the EtOH fed group received daily intragastric gavage of normal saline, and the EtOH fed + LP-HFY09 and EtOH fed + LDSB groups received LP-HFY09 and LDSB at 1.0 × 10^9^ CFU/kg bw, respectively. Three hours later, the mice in these three groups received intragastric gavage of 56° ethanol at 0.13 ml/10 g bw (5.82g/kg); the control group received normal saline treatment twice as the control (Table 1). On the eighth day, the mice were fasted for 12 h, treated accordingly (Table 1), and then euthanized. Blood and liver were collected, and the kidney, heart, and liver were weighed. The percentage of organ weight to bw was used to obtain the organ index (OI).

**Table 1:** Treatment during the experiment.

**Group**	**Day 1 to Day 7**		**Day 8**	
	**10:00**	**13:00**	**10:00**	**12:00**
Control	NS	NS	NS	NS
EtOH fed group	NS	E	NS	E
EtOH fed + LP-HFY09	LAB^a^	E	LAB^a^	E
EtOH fed + LDSB	LAB^b^	E	LAB^b^	E

*NS, normal saline of 0.13 ml/10 g bw; E, 56° ethanol of 0.13 ml/10 g bw*.

a *LAB: 1.0 × 10^9^ CFU/kg bw of Lactobacillus plantarum HFY09, 0.13 ml/10 g bw*.

b *LAB: 1.0 × 10^9^ CFU/kg bw of Lactobacillus delbruechill subsp. bulgaricus, 0.13 ml/10g bw*.

### Morphology of Liver Tissue

Liver tissues were separately soaked in 10% formalin immediately after euthanasia. These tissues were dehydrated, embedded in paraffin, and sliced. The samples were stained with hematoxylin and eosin (H&E) and then, the morphological differences were observed by optical microscopy (BX43, Olympus, Tokyo, Japan).

### Determination of Serum ALT, AST, HAase, and PC III Levels

All blood samples were centrifuged (3,500 rpm, 10 min, 4°C), and serum was then collected into Eppendorf tubes and stored (−80°C) for subsequent analysis. The alanine aminotransferase (ALT) and aspartate aminotransferase (AST) were measured by use of the respective kits (Nanjing Jiancheng Bioengineering Institute, Nanjing, Jiangsu, China). The hyaluronidase (HAase) and precollagen III (PC III) were measured using the respective kits (Shanghai Enzyme-linked Biotechnology Limited Company, Shanghai, China).

### Determination of Serum TNF-α, IL-1β, IL-6, and IL-10

The serum levels of tumor necrosis factor (TNF)-α, interleukin (IL)-1β, IL-6, and IL-10 were measured by the corresponding kits (Shanghai Enzyme-linked Biotechnology Limited Company, Shanghai, China).

### Measurement of Hepatic TC, TG, ADH, ALDH, MDA, SOD, and GSH Levels

Liver tissue was accurately weighed (0.1 g), homogenized (10% homogenate), and centrifuged (4,000 rpm, 4°C, 10 min), to collect the supernatant (17). The hepatic total cholesterol (TC), triglyceride (TG), ADH, acetaldehyde dehydrogenase (ALDH), and malondialdehyde (MDA) were measured using the corresponding kits (Nanjing Jiancheng Bioengineering Institute, Nanjing, Jiangsu, China). Hepatic levels of superoxide dismutase (SOD) and glutathione (GSH) were measured by the corresponding kits (Shanghai Enzyme-linked Biotechnology Limited Company, Shanghai, China).

### Determination of mRNA Expression

The relative expression of target mRNAs was measured by real-time fluorescent quantitative PCR. The total RNA was extracted with TRIzol^TM^ reagent (Thermo Fisher Scientific, Waltham, MA, USA), and the purity and concentration were measured by ultra-microspectrophotometry (Nano-100, All for Life Science, Hangzhou, Zhejiang, China). The RNA, adjusted to 1 μg/μl, was used as templates for reverse transcription PCR (Thermo Fisher Scientific). The PCR reaction was performed with 20-μl reaction volumes using following process: 95°C (60 s); 40 cycles of 95°C (30 s)−65°C (30 s)−72°C (30 s), and then, 95°C (30 s), and 65°C (35 s). C-Jun N-terminal kinase (JNK), extracellular regulated protein kinases (ERK), peroxisome proliferator-activated receptor-α (PPAR-α), SOD1, SOD2, glutathione peroxidase (GSH-Px), nicotinamide adenine dinucleotide phosphate (NADPH), catalase (CAT), and cyclooxygenase-1 (COX1) in liver tissues were measured. GAPDH was used as an internal reference to calculate the relative gene expression using the 2^−Δ*ΔCt*^ method (18).

### Statistical Analysis

The experiments were performed in triplicate to obtain mean values. These data were analyzed by SPSS 22 software (SPSS Inc., Chicago, IL, USA) and are presented as the mean ± standard deviation. Statistical significance (*p* < 0.05) was determined by a one-way analysis of variance using the Duncan multi-range test. All figures were drawn using Origin 8.1 software (OriginLab Corp., Massachusetts, USA).

## Results

### Changes in Body Weights

**Figure 1:**
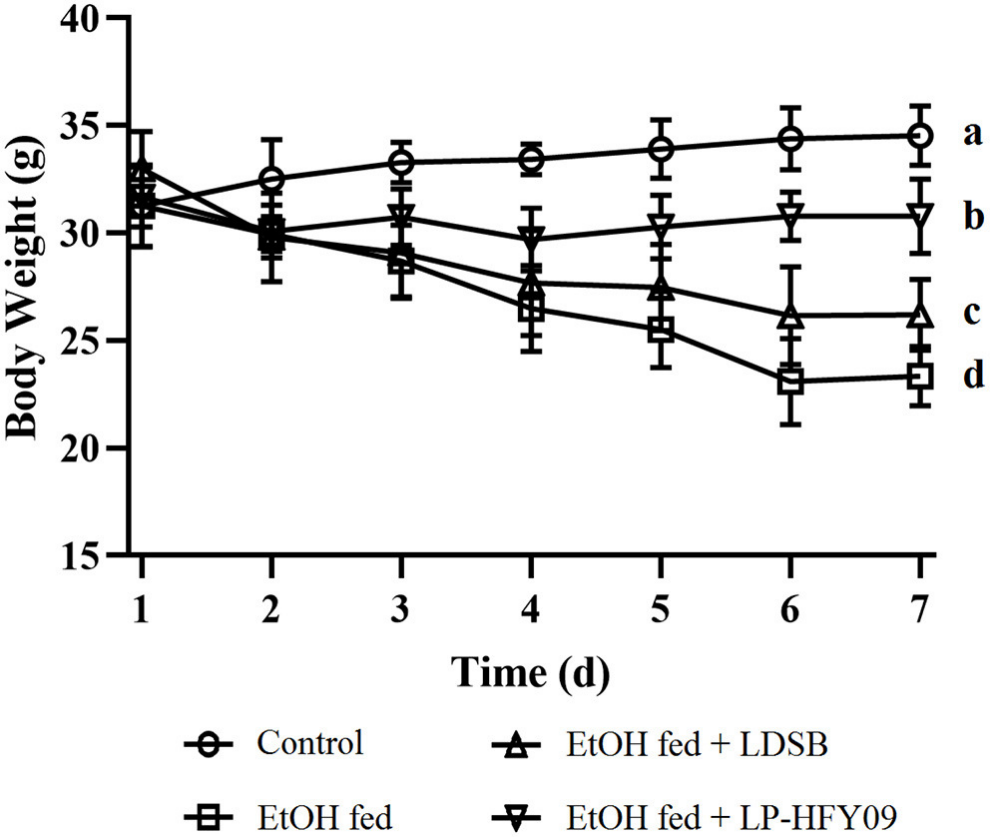
Changes of body weight during the experiment.^a−*d*^ Means significant differences (*p* < 0.05). Control, mice without treatment; EtOH fed, mice treated with 56° ethanol of 0.13 ml/10 g bw; EtOH fed + LDSB, mice treated with 1.0 × 10^9^ CFU/kg of *Lactobacillus delbruechill* subsp. *bulgaricus* before ethanol-treatment; EtOH fed + LP-HFY09, mice treated with 1.0 × 10^9^ CFU/kg of *Lactobacillus plantarum* HFY09 before ethanol-treatment.

At the beginning of the experiment, the bw of the EtOH fed group continuously decreased from 32.99 ± 1.41 to 23.34 ± 1.39 g (*p* < 0.05), while that of the control group maintained a slight increase (*p* > 0.05; Figure 1). At the same time, intervention with LP-HFY09 inhibited and LDSB slowed the losses of bw in the ethanol-induced mice. At the end of the study, the body weights of the EtOH fed + LP-HFY09 group mice were remarkably greater than those in the EtOH fed and EtOH fed + LDSB groups (*p* < 0.05).

### Organ Indexes of the Mice

**Figure 2:**
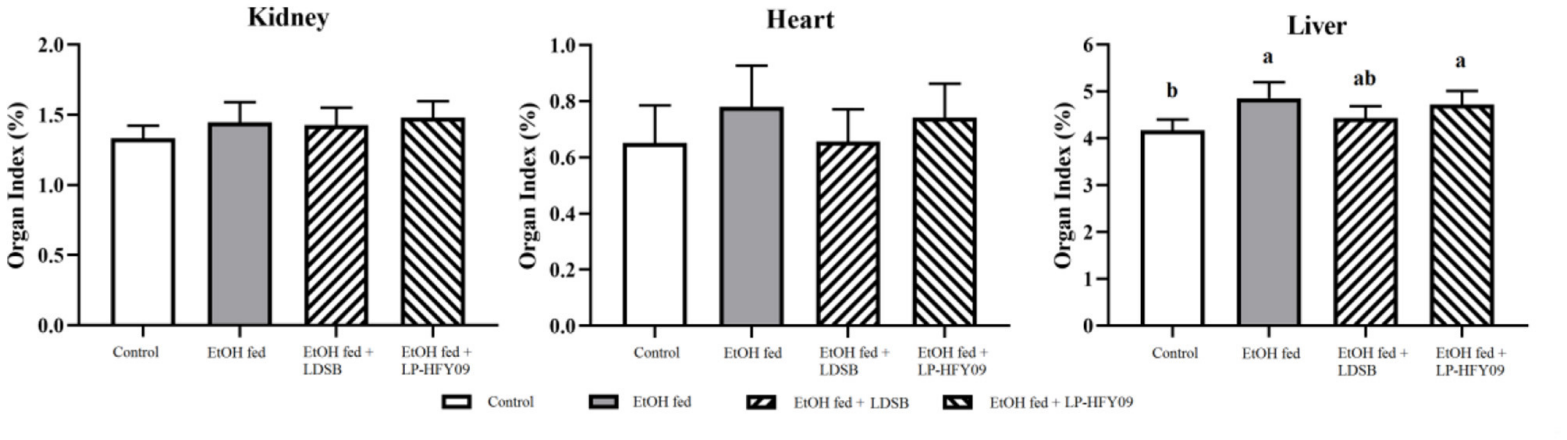
Organ indexes of mice in each group (*n* = 10). ^a, b^In the same sheet means significant differences (*p* < 0.05). Control, mice without treatment; EtOH fed, mice treated with 56° ethanol of 0.13 ml/10 g bw; EtOH fed + LDSB, mice treated with 1.0 × 10^9^ CFU/kg of *Lactobacillus delbruechill* subsp. *bulgaricus* before ethanol-treatment; EtOH fed +LP-HFY09, mice treated with 1.0 × 10^9^ CFU/kg of *Lactobacillus plantarum* HFY09 before ethanol-treatment.

In the model group, the ethanol caused a noticeable increase in the liver index (*p* < 0.05), although there were no remarkable changes in the kidney or heart index (*p* > 0.05; Figure 2). However, the unfavorable effect of the ethanol on the liver was not alleviated by intervention with LP-HFY09 nor LDSB (*p* > 0.05).

### Hepatic Pathological Observation

**Figure 3:**
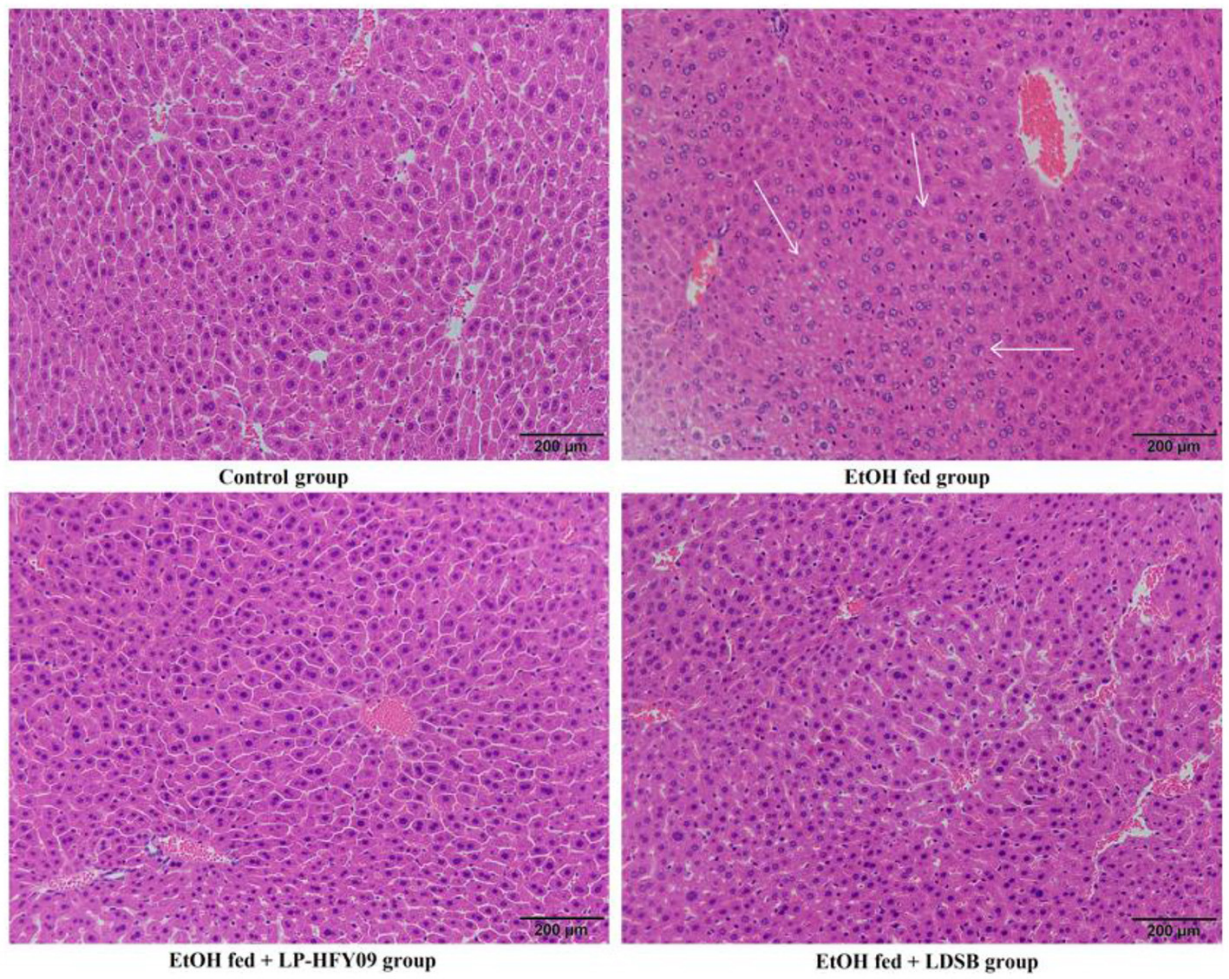
Hematoxylin-eocin (H&E) pathological observation of liver in mice. Magnification 100 × . Control, mice without treatment; EtOH fed, mice treated with 56° ethanol of 0.13 ml/10 g bw; EtOH fed + LDSB, mice treated with 1.0 × 10^9^ CFU/kg of *Lactobacillus delbruechill* subsp. *bulgaricus* before ethanol-treatment; EtOH fed + LP-HFY09, mice treated with 1.0 × 10^9^ CFU/kg of *Lactobacillus plantarum* HFY09 before ethanol-treatment.

As shown in Figure 3, a liver slice from the control group exhibited well-organized structures with uniform cell size, normal lobular architecture with centered nuclei. And the percentage of cell with clear cell boundary was 91.21% (Figure 3). The EtOH fed group, however, showed disorganized structures with unclearly defined cell boundaries (100%), and lack of a regular central vein shape. EtOH fed group also showed signs of inflammatory infiltration, and the area proportion of inflamed cells was 14.55%. At the same time, LP-HFY09 intervention alleviated the above mentioned adverse changes, the liver tissue exhibited morphological characteristics that were similar to those of the control group, and the hepatocyte with normal morphology increased to 47.15%. Some of the morphological characteristics in liver tissue from the EtOH fed + LDSB group were less pathological when compared with the EtOH fed group. But the percentage of cell with clear cell boundary was only 3.95%. Thus, LP-HFY09 might prevent histopathological lesions in the liver that result from ethanol consumption.

### Serum Levels of ALT, AST, HAase, PC III, and Cytokine Indicators

The serum levels of ALT, AST, HAase, PC III, TNF-α, IL-1β, and IL-6 were significantly elevated, and IL-10 was significantly decreased by ethanol when compared to the control group (*p* < 0.05; Table 2). When compared with the EtOH fed group, administration of LDSB and LP-HFY09 significantly decreased the levels of ALT, AST, HAase, PC III, and cytokine indicators (TNF-α, IL-1β, and IL-6), and noticeably increased that of IL-10 (*p* < 0.05). Overall excellent reversals of these disadvantageous changes were observed in response to LP-HFY09 treatment, and even the IL-10 levels in the EtOH fed + LDSB group were significantly higher than those in the EtOH fed + LP-HFY09 group.

**Table 2:** Serum levels of alanine aminotransferase (ALT), aspartic aminotransferase (AST), hyaluronidase (HAase), precollagen III (PC III), interleukin (IL)-1β, IL-6, IL-10, and tumor necrosis factor-α (TNF-α) of mice (*n* = 10).

**Group**	**ALT**	**AST**	**HAase**	**PC III**	**IL-1β**	**IL-6**	**IL-10**	**TNF-α**
	**(U/L)**	**(U/L)**	**(U/L)**	**(U/L)**	**(pg/ml)**	**(pg/ml)**	**(pg/ml)**	**(pg/ml)**
Control	7.87 ± 1.76^c^	13.27 ± 1.18^c^	137 ± 5^d^	4.24 ± 0.31^c^	30.40 ± 1.79^d^	58 ± 4^c^	1175 ± 28^a^	527 ± 21^d^
EtOH fed	21.61 ± 3.61^a^	24.98 ± 3.48^a^	201 ± 14^a^	8.36 ± 0. 34^a^	63.84 ± 2.80^a^	119 ± 7^a^	570± 40^d^	739 ± 35^a^
EtOH fed + LDSB	13.72 ± 1.20^b^	16.71 ± 0.44^b^	174 ± 7^c^	5.33 ± 0.20^b^	43.59 ± 5.44^b^	106 ± 9^b^	826 ± 30^b^	652 ± 35^b^
EtOH fed + LP-HFY09	13.91 ± 1.87^b^	16.97 ± 1.35^b^	155 ± 18^b^	4.56 ± 0.25^c^	36.49 ± 4.90^c^	100 ± 10^b^	720 ± 55^c^	603 ± 33^c^

*Values presented are the mean ± standard deviation (n = 10/group)*.

a−d *In the same column means significant differences (p < 0.05) according to Duncan's multiple range test. Control, mice without treatment; EtOH fed, mice treated with 56° ethanol of 0.13 ml/10 g bw; EtOH fed + LDSB, mice treated with 1.0 × 10^9^ CFU/kg of Lactobacillus delbruechill subsp. bulgaricus before ethanol-treatment; EtOH fed + LP-HFY09, mice treated with 1.0 × 10^9^ CFU/kg of Lactobacillus plantarum HFY09 before ethanol-treatment*.

### Liver Levels of TG, TC, ADH, ALDH, MDA, SOD, and GSH

After 7-day ethanol induction, the accumulation of TC and TG significantly increased 1.94-fold and 0.53-fold (*p* < 0.05), respectively. Enzymatic activities of ADH and ALDH also increased (*p* < 0.05). At the same time, there was a larger elevation in MDA consequent to the sharp drop in liver SOD and GSH (*p* < 0.05). Hepatic parameters indicated that both LAB strains obviously alleviated lipid and oxidation product accumulation (*p* < 0.05), and further enhanced the activity of ADH and ALDH (*p* < 0.05).

### mRNA Expression in Mouse Livers

**Figure 4:**
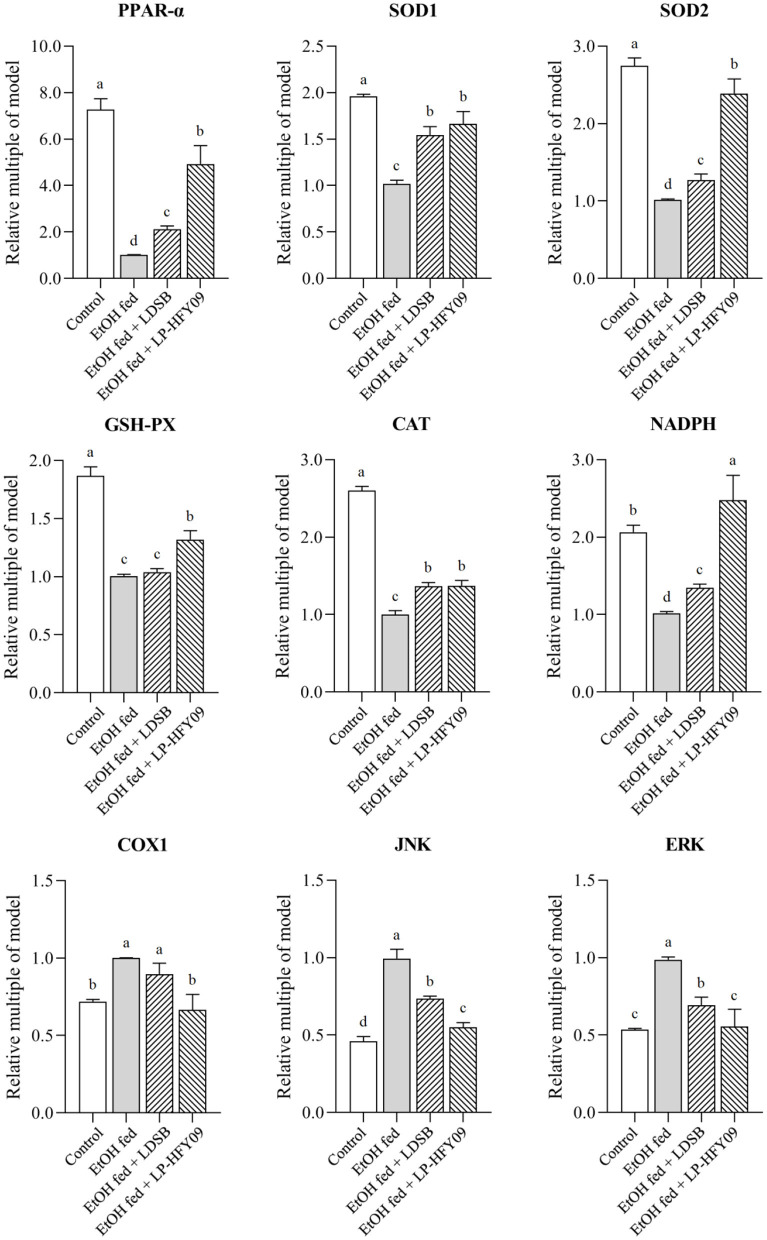
mRNA expressions in hepatic tissue of mice; peroxisome proliferator-activated receptor alpha (PPAR-α), superoxide dismutase 1 (SOD1), superoxide dismutase 2 (SOD2), and glutathione-peroxidase (GSH-PX), catalase (CAT), nicotinamide adenine dinucleotide phosphate (NADPH), cyclooxygenase-1 (COX1), c-Jun N-terminal kinase (JNK), and extracellular regulated protein kinases (ERK). ^a−*d*^ The different letters mean that there are significant differences (*p* < 0.05) between every two groups, and the same letters mean that there is no significant difference (*p* > 0.05) between every two groups according to Duncan's multiple range test. Control, mice without treatment; EtOH fed, mice treated with 56° ethanol of 0.13 ml/10 g bw; EtOH fed + LDSB, mice treated with 1.0 × 10^9^ CFU/kg of *Lactobacillus delbruechill* subsp. *bulgaricus* before ethanol-treatment; EtOH fed + LP-HFY09, mice treated with 1.0 × 10^9^ CFU/kg of *Lactobacillus plantarum* HFY09 before ethanol-treatment.

Figure 4 illustrates that *PPAR-*α, *SOD1, SOD2, GSH-PX, CAT*, and *NADPH* in the EtOH fed group were noticeably downregulated by ethanol, while *JNK, ERK*, and *COX1* were upregulated (*p* < 0.05). After preliminary treatment with LP-HFY09, all these genes were significantly regulated (*p* < 0.05), with partial recovery when compared with the control group. Moreover, the expression of *NADPH* in EtOH fed + LP-HFY09 group was recovered and significantly higher than that in control group (*p* < 0.05). In the EtOH fed +LDSB group, the changes induced by ethanol were also attenuated to some extent (*p* < 0.05). When the LAB interventions were compared, it was observed that there were no significant differences in the regulation of *SOD1* or *CAT* (*p* > 0.05). *Lactobacillus plantarum* HFY09 partly ameliorated the effects of ethanol-induced liver-injury on mRNA expression of some antioxidase and oxidative metabolism-related genes.

## Discussion

Consumption of probiotics is increasing around the world because they promote health. Probiotics have been defined by the Food and Agriculture Organization of the United Nations and World Health Organization (FAO/WHO) (2006) as a group of living microorganisms that can confer beneficial health effects with adequate intake (19). Probiotics have been widely used in daily life in the form of ordinary foods as well as lyophilized compounds and pills, and constitute a growing multi-billion-dollar industry (20, 21). Some studies have noted that LAB probiotics such as *L. plantarum* C88 (18), *Streptococcus thermophilus* GRX02 (22), *Pediococcus pentosaceus* LI05 (23), *Lactobacillus rhamnosus* GG (24), and *L. rhamnosus* CCFM1107 (25) prevent acute or chronic alcoholic injury of the liver. Studies have indicated that the mechanisms of liver protection by probiotics mainly include repair of the intestinal mucosa (26), reduced TNF-α levels (27, 28), and enhanced antioxidant capacity (29). Our preliminary experiment showed the antioxygenation of newly isolated LP-HFY09 *in vitro*, indicating that the strain might mitigate the injury caused by alcohol.

The liver is a vital organ that perform many functions, including lipid metabolism and immunity (30). Alcohol causes oxidative stress and inflammation, which reduce hepatocyte function and increase fat accumulation, and heavy drinking can induce liver fibrosis (31). Moreover, alcohol consumption also decreases appetite, as it was observed in this study that the daily fodder intake of the ethanol-treated mice was 2–3 g lower as compared to that of the mice in control group. Consequently, the body weights of the mice in the EtOH fed group significantly decreased.

The common probiotic LDSB was used for comparison with LP-HFY09, and it was observed that weight loss did not occur in mice treated with LP-HFY09 or LDSB. Alanine aminotransferase and aspartate aminotransferase are important enzymes reflecting liver function, and they are released into the bloodstream during hepatocytic injury (32). Hyaluronidase and precollagen III are sensitive indexes of liver fibrosis and also accurately reflect the amount of damage to liver cells. Increased serum levels of ALT, AST, HAase, and PC III in the EtOH fed group proved that alcohol damaged the liver and degraded its functions, and in the EtOH fed + LAB groups, there was a significant reduction in adverse effects, due to those interventions (Table 2). The increased levels of TC and TG also suggested that damage occurred to the lipid metabolic function, resulting in the accumulation of lipid, while they were decreased by the administration of LAB (Table 3).

**Table 3:** Liver levels of triglyceride (TG), total cholesterol (TC), alcohol dehydrogenase (ADH), acetaldehyde dehydrogenase (ALDH), superoxide dismutase (SOD), and glutathione (GSH) in hepatic tissue of mice (*n* = 10).

**Group**	**TG**	**TC**	**ADH**	**ALDH**	**MDA**	**SOD**	**GSH**
	**(mmol/g prot)**	**(mmol/g prot)**	**(U/mg prot)**	**(U/mg prot)**	**(nmol/mg prot)**	**(U/mg prot)**	**(μmol/g prot)**
Control	0.66 ± 0.08^c^	3.62 ± 0.10^c^	21.39 ± 2.59^d^	11.24 ± 0.89^d^	0.97 ± 0.19^d^	729 ± 37^a^	25.86 ± 4.26^a^
EtOH fed	1.94 ± 0.30^a^	5.54 ± 0.42^a^	40.08 ± 3.34^c^	13.91 ± 1.19^c^	3.45 ± 0.26^a^	493 ± 26^d^	10.76 ± 1.27^c^
EtOH fed + LDSB	1.28 ± 0.23^b^	4.62 ± 0.24^b^	54.58 ± 3.42^b^	17.65 ± 0.84^b^	2.42 ± 0.24^b^	580 ± 39^c^	16.02 ± 0.53^b^
EtOH fed + LP-HFY09	1.03 ± 0.27^b^	3.69 ± 0.52^c^	65.56 ± 4.55^a^	21.01 ± 0.80^a^	1.64 ± 0.32^c^	668 ± 28^b^	18.66 ± 4.89^b^

*Values presented are the mean ± standard deviation (n = 10/group)*.

a−d *In the same column means significant differences (p < 0.05) according to Duncan's multiple range test. Control, mice without treatment; EtOH fed, mice treated with 56° ethanol of 0.13 ml/10 g bw; EtOH fed + LDSB, mice treated with 1.0 × 10^9^ CFU/kg of Lactobacillus delbruechill subsp. bulgaricus before ethanol-treatment; EtOH fed + LP-HFY09, mice treated with 1.0 × 10^9^ CFU/kg of Lactobacillus plantarum HFY09 before ethanol-treatment*.

When inflammation occurs, IL-6 is initially produced, and the degree of elevation is consistent with the severity. Tumor necrosis factor-α regulates immune responses by inducing cytokine production (33), while IL-1β increases the adhesive capacity of cytokines, and IL-10 regulates immune responses and inhibits inflammation to protect organs and tissues (34, 35). In this study, LP-HFY09 treatment prevented liver damage and inflammation by significantly decreasing the serum IL-6 and TNF-α, and increased IL-1β and IL-10 (Table 2), thus alleviating the histopathological changes in the liver tissue (Figure 3).

Alcohol dehydrogenase is a crucial enzyme for ethanol metabolism that is mainly produced in the liver, and it converts ethanol to acetaldehyde. Aldehyde dehydrogenase also plays an important role in metabolizing endogenous and exogenous aldehydes to carboxylic acids (36). Both ADH and ALDH influence alcoholism, and their activities are induced by ethanol, which was observed in the EtOH fed group by increasing activities of these two enzymes. The enzymatic activities of the EtOH fed + LAB groups were further increased in comparison to the EtOH fed group (Table 3). Some alcoholic detoxification tablets work by increasing the activity of ADH and ALDH, and LP-HFY09 and LDSB also exhibit the ability to quickly counteract the effects of alcohol.

Oxidative stress is an imbalance between the production and degradation of ROS. Reactive oxygen species are produced at each step of alcohol metabolism. Excess acetaldehyde entering the bloodstream also will be converted to superoxide by perxanthine oxidase. Malondialdehyde is the final product of free radical-mediated lipid peroxidation, and is commonly used as a marker of oxidative stress. Superoxide dismutase is a key scavenger of ROS that converts superoxide to oxygen and hydrogen peroxide in aerobic organisms, and GSH is also an important antioxidant component (37). The ethanol in alcoholic beverages is absorbed by the gastrointestinal tract, and 90% of that is oxygenated and metabolized in the hepatic cells, generating large amounts of free radicals (37). Both the decreased levels of SOD and GSH, and increased level of MDA in the EtOH fed group indicated the amount of oxidant stress induced by ethanol (Figure 3). Treatment with LP-HFY09 or LDSB before gavage administration of ethanol significantly relieved oxidative stress by increasing the activity of SOD and the amount of GSH, and thus sharply decreased the MDA levels in liver tissue. *Lactobacillus plantarum* HFY09, however, exhibited greater efficiency in counteracting antioxidant stress than that of LDSB (*p* < 0.05).

Proliferator-activated receptors with three subtypes are widely recognized as ligand-induced nuclear receptors that are responsible for various biological effects. Among these subtypes, PPAR-α is highly expressed in liver tissues, is closely related to lipid metabolism (fatty acid β-oxidation), and participates in the respiratory chain of oxidation (38, 39). The magnitude of change in *PPAR-*α expression is related to the amount of damage incurred by the liver. As illustrated in Figure 3 and Table 2, the morphology and function of the liver in the EtOH fed group was most damaged by alcohol, and this consequently depressed the level of PPAR-α, which affected lipid metabolism and resulted in increased lipid accumulation.

It is known that alcohol can cause oxidative stress and generate ROS. The mitochondrial respiratory chain is another source of increased levels of ROS after ethanol intake (40). We observed that the expression of antioxidant enzymes (SOD1, SOD2, CAT, and GSH-PX) and coenzyme (NADPH) was downregulated by ethanol. This downregulation reduces the antioxidant enzyme activities and the ability to neutralize ROS, which leads to oxidative stress. SOD, including SOD1 and SOD2, is the first detoxification enzyme that neutralizes superoxide radicals. SOD2, which is located in the mitochondrial matrix, neutralizes superoxide radicals by catalyzing the dismutation of superoxide, and then produces H_2_O_2_. The harmful H_2_O_2_ can then be converted into O_2_ and H_2_ by SOD1 (41). And in the study, both the expression of SOD mRNA and the concentration of hepatic SOD increased after being intervened by potential probiotic strain.

The harmful H_2_O_2_ also can be degraded into O_2_ and H_2_O by CAT and GSH-Px (42). Catalase exists in the cytoplasm and can reduce fat accumulation without oxidative damage and control the peroxisomal H_2_O_2_ that degrades hepatic fatty acids, thus maintaining the optimal metabolic balance (43). Nicotinamide adenine dinucleotide phosphate acts as a hydrogen transmitter in many chemical reactions (e.g., electron transport chain) and is an essential cofactor in the reduction of GSH to its reduced form (44). The level of reduced GSH fluctuates with the content of NADPH, and the decreased NADPH leads to increased ROS and results in oxidative damage to tissues. The decreased levels of SOD1, SOD2, GSH-Px, CAT, and NADPH indicated that there were lower antioxidant levels in alcohol-fed mice, and explain the higher levels of MDA in the livers of the EtOH fed group mice. It was observed that intervention by LP-HFY09 and LDSB increased all the enzymes and coenzyme mentioned above. Moreover, greater improvement was observed as a result of LP-HFY09 treatment as compared to LDSB, and the level of NADPH was significantly higher than that of the control group (*p* < 0.05).

The cyclooxygenases (COXs), including two types of isozymes (COX1 and COX2), can be induced by many stimulants, such as proinflammatory stimulators and endotoxins. COX1 is associated with inflammation, and is more responsive than COX2 (45). Thus, COX1 is now considered as a target for inflammatory treatment. In this study, the mice in the EtOH fed group exhibited the highest COX1 level, but it was decreased by the intervention of LP-HFY09; the inflammatory factors (IL) exhibited the same trends (Table 2). These results indicated that LP-HFY09 reduced the production of stimulants, thus alleviated the inflammatory state in the liver.

c-Jun N-terminal kinase, also called stress-activated protein kinase (SAPK), and ERK are important members of the mitogen-activated protein kinase (MAPK) family, which plays an important role in the information transfer from outside of the cell to the nucleus. Jun N-terminal kinase and extracellular regulated protein kinases are easily activated by TNF, IL, and ROS, as well as metabolic syndrome, and both play important roles in various processes of physiology and pathology (46). Previous studies indicated that alcohol intake led to the release of proinflammatory factors, further activation of JNK, and acceleration of ALD (47), although reduced hepatic injury occurred in JNK knockout mice (48). Liver fibrosis can be induced by alcohol, which is responsible for the excessive deposition of extracellular matrix (ECM), especially collagen, and is closely related to the expression of ERK that significantly affects the production and degradation of the ECM (49, 50). The expression of *JNK* and *ERK* was significantly upregulated in the EtOH fed group (Figure 4), along with increased levels of TNF-α, IL-1β, and IL-6, as well as decreased levels of SOD and GSH (Tables 2, 3), that were significantly downregulated by LP-HFY09.

In our study, LP-HFY09 significantly upregulated the mRNA expression of *PPAR*α, *SOD1, SOD2, GSH-PX, CAT*, and *NADPH*, and downregulated the expression of *COX1, JNK*, and *ERK*. *Lactobacillus plantarum* HFY09 administration resulted in weight loss, reduced liver injury, increased liver function, and decreased inflammation and lipid accumulation. *Lactobacillus plantarum* HFY09 also significantly increased the antioxidant capacity. It exerts all these actions by regulating the expression of antioxidant enzymes.

## Conclusions

This investigation showed that administration of LP-HFY09 (1.0 × 10^9^ CFU/kg bw) inhibits ethanol-induced loss weight and decreased the inflammatory response. *Lactobacillus plantarum* HFY09 also increased antioxidant action and enhanced the prevention ethanol-damage capacity. Additionally, the intervention effect of LP-HFY09 was significantly greater than that of commercially used *Lactobacillus bulgaricus*. This investigation indicates that there is great potential for LP-HFY09 to be used as a probiotic that can maintain hepatocyte morphology and relieve the negative influences of ethanol on the liver. Further mechanism studies will be performed to determine the extent of the probiotic effects of LP-HFY09.

## Data Availability Statement

The original contributions presented in the study are included in the article/supplementary material, further inquiries can be directed to the corresponding author/s.

## Ethics Statement

The protocol for these experiments was approved by the Ethics Committee of Chongqing Collaborative Innovation Center for Functional Food (202006003B), Chongqing, China. The experimental process was in accordance with 2010/63/EU directive.

## Author Contributions

XZha and WL designed research and supervised the study. YG and JT wrote the manuscript and interpreted data. XZho, XL, and YP analyzed and interpreted data. All authors agree to be held accountable for the content therein and approve the final version of the manuscript.

## Conflict of Interest

The authors declare that the research was conducted in the absence of any commercial or financial relationships that could be construed as a potential conflict of interest.
